# Motion Artifact Reduction in Electrocardiogram Signals Through a Redundant Denoising Independent Component Analysis Method for Wearable Health Care Monitoring Systems: Algorithm Development and Validation

**DOI:** 10.2196/40826

**Published:** 2022-11-25

**Authors:** Fabian Andres Castaño Usuga, Christian Gissel, Alher Mauricio Hernández

**Affiliations:** 1 Bioinstrumentation and Clinical Engineering Research Group, Bioengineering Department, Engineering Faculty Universidad de Antioquia Medellín Colombia; 2 Department of Health Economics Justus Liebig University Giessen Giessen Germany

**Keywords:** signal denoising, motion artifacts, biomedical signal processing, electrocardiogram, ECG, biomedical monitoring, home health care

## Abstract

**Background:**

The quest for improved diagnosis and treatment in home health care models has led to the development of wearable medical devices for remote vital signs monitoring. An accurate signal and a high diagnostic yield are critical for the cost-effectiveness of wearable health care monitoring systems and their widespread application in resource-constrained environments. Despite technological advances, the information acquired by these devices can be contaminated by motion artifacts (MA) leading to misdiagnosis or repeated procedures with increases in associated costs. This makes it necessary to develop methods to improve the quality of the signal acquired by these devices.

**Objective:**

We aimed to present a novel method for electrocardiogram (ECG) signal denoising to reduce MA. We aimed to analyze the method’s performance and to compare its performance to that of existing approaches.

**Methods:**

We present the novel Redundant denoising Independent Component Analysis method for ECG signal denoising based on the redundant and simultaneous acquisition of ECG signals and movement information, multichannel processing, and performance assessment considering the information contained in the signal waveform. The method is based on data including ECG signals from the patient’s chest and back, the acquisition of triaxial movement signals from inertial measurement units, a reference signal synthesized from an autoregressive model, and the separation of interest and noise sources through multichannel independent component analysis.

**Results:**

The proposed method significantly reduced MA, showing better performance and introducing a smaller distortion in the interest signal compared with other methods. Finally, the performance of the proposed method was compared to that of wavelet shrinkage and wavelet independent component analysis through the assessment of signal-to-noise ratio, dynamic time warping, and a proposed index based on the signal waveform evaluation with an ensemble average ECG.

**Conclusions:**

Our novel ECG denoising method is a contribution to converting wearable devices into medical monitoring tools that can be used to support the remote diagnosis and monitoring of cardiovascular diseases. A more accurate signal substantially improves the diagnostic yield of wearable devices. A better yield improves the devices’ cost-effectiveness and contributes to their widespread application.

## Introduction

### Problem Statement

Digital health provides the opportunity to combat pandemics, to deliver health care in remote regions, and to reduce the carbon footprint of health care delivery. Telehealth and remote monitoring, particularly in patients’ homes, has become an important option due to problems associated with keeping patients in hospitals and care centers for extended periods: the increase in the probability of acquiring nosocomial infections [1]; the deficiency in medical infrastructure to meet the demand of patients [2]; the increase in therapeutic dependence by older adult patients [3]; and the increase in hospitalization costs. These issues have led to a search for alternatives in medical care such as home health care.

This has led to an increase in the development of remote monitoring technologies of patients’ vital signs to improve the medical diagnosis [4,5]. Wearable devices for monitoring vital signs have become a powerful tool to improve health services and to implement home health care models [6,7]. These will allow to acquire vital signs of patients in daily environments while they carry out their activities in a normal way, allowing to obtain complementary information to improve the medical diagnosis, to constantly monitor the patient’s condition, and to improve their treatments [7,8].

A major challenge for the diffusion of digital health technologies lies in the signal quality of sensors for remote diagnosis and monitoring of patients, including electrocardiogram (ECG) signals of moving patients, which require the denoising of motion artifacts (MA). ECG is an important diagnostic technique for application in wearable devices, owing to the amount of information contained in the acquired waveform, distributed in different peaks and undulations called segments (P, Q, R, S, T, and U). The P segment represents atrial depolarization, the QRS complex represents ventricular depolarization and atrial repolarization, the isoelectric ST segment is the time when both ventricles are completely depolarized, the T segment represents ventricular repolarization, and the U segment represents papillary muscle repolarization [9,10]. Each segment is characterized by its unique shape, amplitude, duration, and time of occurrence, allowing to identify the way in which the electrical impulse is conducted through the heart muscle [10].

### State of the Art

Newer devices have been developed to identify cardiovascular diseases in early stages (asymptomatic). Some of them are external loop recorders, implantable loop recorders, and Zio patches as well as wearable ECGs. Similar to traditional Holter, they share a sensitivity to artifacts [11], which leads to repeated ECG monitoring with cost increases in 11.1% of cases [9]. In the case of wearable ECGs, the information provided by these devices is not considered for clinical use owing to the contamination by different noise sources such as power line interference, baseline wander, and MA that have nonlinear, nonstationary, and unpredictable character, as well as ECG bandwidth overlaps [12-14]. In addition, the effects of daily life movements on the signal are difficult to predict, which makes the devices’ validation for medical use in home health care and outdoor conditions even more difficult. This motivates the development of techniques that allow the reduction of interference in the ECG signal.

Previously, research has been conducted to develop techniques that solve the problem of combined interference of MA, baseline wander, and power line interference in ECG signals [15]. Performance assessments of denoising techniques such as wavelet shrinkage (WS), empirical mode decomposition (EMD), wavelet independent component analysis (WICA), and EMD independent component analysis (ICA) have been performed. These methods present problems, although some of these work in the denoising of synthetic signals when working with signals from patients in movement. One of them is that their signal databases only consider a single source of information (ECG signal) to perform signal denoising, which makes it difficult to acquire the dynamics introduced by movement and significantly affects the performance of artifact reduction methods. However, it was found that depending on the segment of the ECG signal to be preserved, it is possible to use a specific denoising technique for that segment [16].

In recent years, significant advances in the development of techniques for feature extraction from cardiovascular signals in wearable monitoring have been made. The presence of MA has been identified as a significant source of noise in signal acquisition, masking information about the physiological process and leading to misdiagnosis. The MA reduction problem is still addressed in different ways as proposed by Yang and Tavassolian [17], where it is possible to obtain cardiovascular parameters from the seismocardiography signal analysis; they used the ICA on the inertial signals acquired from inertial measurement units (IMUs) and used the ECG and photoplethysmography (PPG) signals as reference signals. An and Stylios [18] evaluated conventional filtering methods using finite impulse response, infinite impulse response, moving average, moving median filters, and advanced decomposition methods such as wavelet, EMD, and adaptive filters to compare their performance. They found that all these methods have their limitations, but the best method was considered to be the adaptive filter. However, it depends on a good selection of the reference signal and still introduces distortion to the signal [18,19].

Other approaches use adaptive noise signal detection, EMD, or wavelet decomposition of the signal of interest and dynamic time warping (DTW) component selection to reduce baseline wandering and high-frequency noise, and they achieved signal improvements of up to 25% [11,20,21]. On the other hand, the electrode configuration and its interaction with the skin had been evaluated to determine the impedance variation and the noise introduction in the signal acquisition [22-24]. Many authors agree that the way to approach the problem is through the use of signal decomposition methods such as wavelets, EMD, and ICA, among others [25-27]. In addition, including multiple sources of information such as pressure signals, PPG and movement are essential to estimate the physiological parameters of interest [28].

### Study Objectives

Although some of these methods use the acquisition of multiple signals as other sources of information for the determination of cardiovascular parameters, few of them are focused on evaluating the recovery of the waveform of the ECG signal. Similarly, dealing with the acquisition of multiple physical magnitudes such as pressure or PPG through a single channel does not allow confirmation of the correct denoising of the signal of interest. The method proposed in this paper allows the redundant and simultaneous acquisition of ECG signals and movement signals to obtain more complete information of the physiological process masked by the movement artifacts.

It has been observed that the correct location of the electrodes on the volume conductor of the patient has a great influence on the result of the ECG, to the point that a bad location of these can lead to diagnostic mistakes [29,30]. However, it has been identified that certain modifications in the signal acquisition hardware, such as adding additional leads in the back and taking information of the movement of the person, allow obtaining additional information of the signal of interest [16,31]. In this study, additional electrodes were added to the acquisition hardware of the ECG signal in the chest and back of the volunteers. This represents a novel method for the acquisition of the signals in a redundant and simultaneous way to improve the denoising, decreasing the distortion introduced in the MA reduction process.

The distortion concept in the context of biomedical signal processing refers to the change in the natural shape of the signal due to external processes or disturbances, which include changes in the amplitude, duration, or time of occurrence of the segments that compose the signal and lead to loss of information. The concept of redundant measurement refers to the acquisition of information from the same interest source from ≥2 different measurement points, with the purpose of preventing the loss of information or increasing the sources of information of interest, as is true in this study. Redundant measurements are performed simultaneously to ensure that the information acquired from the different measurement points is synchronized, which is defined as multichannel synchrony.

Further improvement of noise and MA reduction is critical to achieve an optimal diagnostic yield with wearable health care monitoring systems. The diagnostic yield will be an important determinant of the devices’ cost-effectiveness [32]. Only with a reliable diagnosis of specific cardiac arrythmias such as atrial fibrillation will the devices’ cost-effectiveness allow widespread application, even in resource-constrained environments [33].

## Methods

### Overview

This paper presents a novel method for the reduction of noise and MA in ECG signals from walking individuals in ambulatory vital signs monitoring applications. We have introduced a new method called Redundant denoising Independent Component Analysis (Rd-ICA). It is based on (1) redundant and simultaneous measurement of ECG signals in the chest and back; (2) acquisition of triaxial movement signals from IMUs; (3) a reference signal synthesized from an autoregressive model, which considers the features of a resting ECG signal obtained through ensemble average (EA) ECG [9,16]; and (4) separation of interest sources and noise sources through multichannel ICA. After the separation of the signals, the identification of the ECG signal is made through the comparison of the components with the synthesized reference ECG signal.

The performance of this method is tested with a database composed of data sets of movement signals and ECG signals acquired in the chest and the back from healthy volunteers in conditions of rest and movement. In addition, the performance of the Rd-ICA method is compared with the performance presented by state-of-the-art denoising methods such as the WS method and the WICA method. The calculation of performance indexes is performed with indexes such as the signal-to-noise ratio (SNR), the DTW, and a proposed index defined as weighted distortion assessment (WDA). It measures the characteristics of the shape of wave found with the EA ECG method.

This section shows the protocol for recording ECG signals, presents some previous state-of-the-art methods for ECG signal denoising, and finally shows the proposed Rd-ICA method. In this work, the comparison of the methods’ performance was also carried out. A new index based on the signal distortion characterization through the EA ECG has been proposed.

### Register Protocol

A database of ECG signals acquired from a population of 20 healthy volunteers aged, on average, 26.3 (SD 5.7) years with an average BMI of 24.4 (SD 4.8) kg/m^2^ was registered. Database registration was performed for bipolar leads DI, DII, and DIII in the chest and the back and the triaxial movement signal of the volunteer. The experiment was divided into 3 stages: (1) rest before movement, (2) controlled movement in laboratory conditions, and (3) rest after movement. Each volunteer was asked in the first stage to remain at rest for 5 minutes, which led to the acquisition of reference ECG and motion signals. In the second stage, each volunteer was asked to perform a walk at a normal travel speed of 4.2 (SD 0.8) km/h for 5 minutes, which produces contamination that masks and distorts the ECG signal significantly. In the third stage, each volunteer was asked again to be at rest for 5 minutes. This protocol was performed to obtain a database of ECG signals contaminated with MA composed of redundant and simultaneous ECG signals acquired in the chest and the back, also with the movement of the volunteer registered through IMUs [24]. ECG signals were acquired at a sampling frequency of 250 Hz and 24-bit resolution [34]. Informed consent was obtained from all participants involved in the study.

To acquire signals, a custom wearable device was used that performs the acquisition of the signals of ECG; PPG; and noninvasive blood pressure that is redundant, simultaneous, and synchronized [31,35]. The ECG is acquired on the volunteer’s chest and back, and an IMU is included on each ECG lead to record the inertial activity and movement. It similarly occurs for the PPG signal that is acquired both in the left wrist and the right wrist and for the noninvasive blood pressure signal that is recorded in both arms through the oscillometric method [8]. The IMUs allow acquiring the inertial activity and movement to analyze the nonlinear dynamics of artifact contamination on the signal.

All the signals are recorded simultaneously and synchronized in time to guarantee signal redundancy and the possibility of applying the Rd-ICA method. For the ECG signal, Ag/AgCl electrodes were used, which have proven to be the ones with the least interference in recording of the signals due to their correct coupling with the skin.

**Figure 1:**
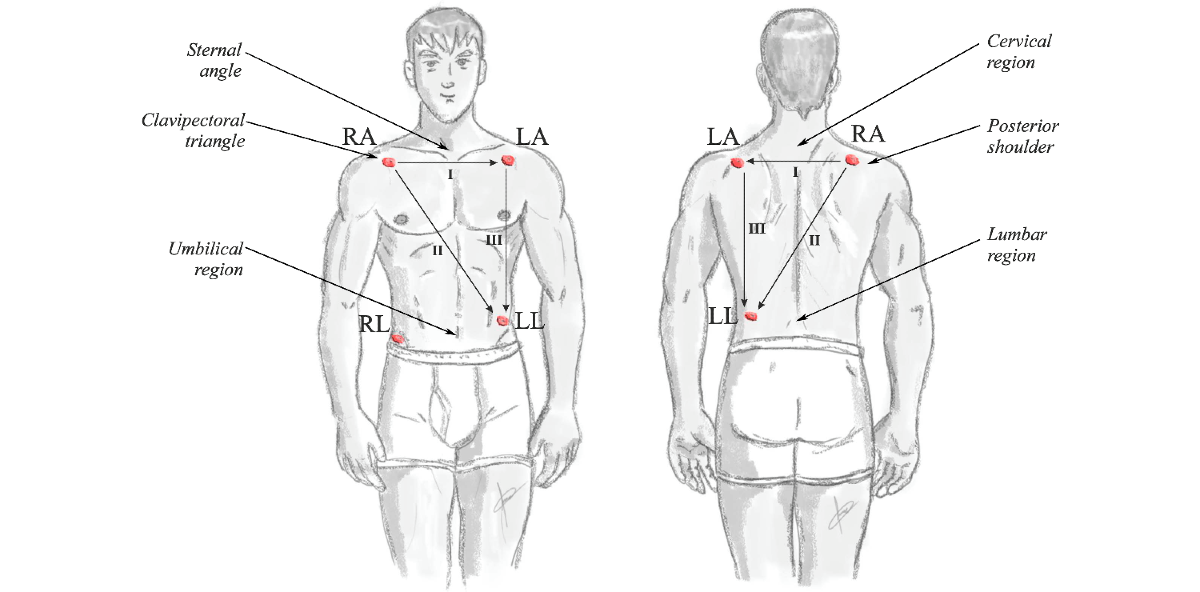
Electrodes' distribution for the acquisition of electrocardiogram signals according to the Einthoven triangle in the Mason-Likar [38] method over the chest and back of the volunteer. LA: left arm; LL: left leg; RA: right arm; RL: right leg.

In clinical ECG, it is common to place the electrodes on the arms such as the left and right wrists and the left foot under the Einthoven triangle model. In long-term examinations such as Holters, it is common to use the positioning of the electrodes according to the Einthoven triangle under the Mason-Likar [36] method in which the electrodes are located on the person’s chest. This model is the most frequently used in ECG wearable monitoring [36,37]. Signals were acquired through the connection of an electrophysiological signal recording equipment to acquire the biopotentials in the torso and back of 20 volunteers as previously validated [24,31]. A group of sensors were placed in the location proposed by the Einthoven triangle in the Mason-Likar [36] method in the chest. These locations were interpolated on the back of the volunteer, considering anthropometric locations [38]. In addition, the acquisition of triaxial movement signals was performed through an IMU located in the electrophysiological sensors. It should be noted that the acquisition of electrophysiological signals in the chest and back was performed redundantly and simultaneously, synchronized with the movement signals. Figure 1 shows the distribution of electrodes in the chest and back of the volunteer.

### Techniques to Reduce MA

The most frequently used techniques to reduce MA have previously been described in detail, including WS, ICA, and WICA [17].

#### Wavelet Shrinkage

The discrete wavelet transform (DWT) allows to represent a signal as a set of waves through 2 types of functions called mother wavelet and father wavelet, which contain high and low frequency information [39-41]. DWT is a denoising method for ECG signals in a process known as multiresolution analysis [42], where the signal is decomposed in different levels through Mallat tree decomposition and Daubechies 8 mother wavelet selection [43-45]. Then, the thresholding method reduces the noise components with the RiskShrink algorithm from the signal before the reconstruction through inverse DWT is performed [46,47]. In this study, the WS method was applied on each acquired derivation in the chest of volunteers to perform the denoising of ECG signals to compare the performance with the proposed method [48].

#### Independent Component Analysis

Some measured signals can be considered a linear mixture of information from independent sources, such as artifacts, noise, and interest signals. It is possible to separate these sources with the ICA method [49,50]. To apply the ICA, it is necessary to have a set of observations x = (*x_1_, x_2_,...x_m_*)*^T^* taken from *m* sensors. The observations are modeled as the linear combination of a set of signals s = (*s_1_, s_2_,...s_n_*)*^T^*, as is described by the mixing model (equation 1) [49,51].



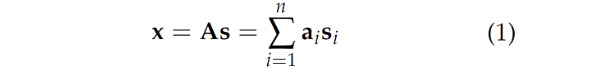



Where the mixing matrix A = (a_1_, a_2_,...a_n_) that has a size of *m* × *n* and *a_i_* are the vectors of the mixture.

To apply the ICA, it is necessary to assume that the sources’ signals are independent; just one component has a Gaussian distribution at most; and the number of sensors must be equal to the number of independent sources (*m* = *n*) [51]. Through the ICA method, the separation matrix w = (w_*ij*_)_(*n* x *n*)_ and the *n* separate signals Y = (*y_1_, y_2_,...y_n_*)*^T^* can be obtained (equation 2).


Y = Wx = WAs = Gs (2)

Some of the sources are noise sources and should not be considered in the model to perform the denoising. Then, some elements of the separation matrix W must be forced to 0 to reduce the influence of the artifacts on the interest signal [51].

#### WICA Denoising

As previously described, some extensions of the ICA using decomposition techniques such as DWT have been proposed to denoise physiological signals [17]. The WICA performs the DWT decomposition to obtain multichannel signals from a single-channel signal before applying the ICA method [52]. The process is outlined in the form of an algorithm in Textbox 1.

In this study, the WICA method was applied to the ECG signals acquired in the chest of the volunteers to evaluate its performance. The selection of noise sources or artifacts is performed conventionally through visual inspection as is proposed by other studies, which is one of the main drawbacks of this method [51,52].

**Wavelet independent component analysis (ICA) algorithm.**

**Algorithm**

1. Select the mother wavelet and the order of the wavelet transform.
2. Apply the wavelet decomposition (discrete wavelet transform [DWT]) to generate the input matrix for the ICA algorithm.
3. Apply the ICA method to the set of wavelet components and derive the corresponding mixing (A) and demixing (W) matrices.
4. Select the sources of interest, force the others to 0, and multiply this selection with the mixing matrix (A) to back-reconstruct their appearance in the set of wavelet components.
5. Apply the inverse DWT over the new set of wavelet components to back-reconstruct the enhanced signal.

#### EA Electrocardiogram

The EA ECG allows the characterization of the ECG signal through the measurement of segments’ features that compose the ECG signal (P, Q, R, S, T, and U). This method has been used to evaluate the ECG signal distortion introduced by MA. It allows to evaluate the performance of denoising methods quantitatively considering the waveform of the signal. This is done by finding the average pattern of a signal that has a periodically repeated waveform, which is the case for the ECG signal [16].

In the EA ECG computing process, it was necessary to select a fiducial point on the standard waveform, which was the reference in time to synchronize the signal waveforms. The R peak was selected because it has the maximum amplitude in waveform. On the other hand, the size in time or in samples that have the standard waveform was determined to perform the partition of the signal, the synchronization through the fiducial points and the averaging of the signals. This time was determined as the time elapsed between 2 consecutive R peaks and corresponds with the heart rate.

In this study, the method was used first to perform the characterization of the ECG signals acquired at rest as a reference. In addition, the method was used to measure the performance by state-of-the-art denoising methods and the proposed Rd-ICA method. Furthermore, the features obtained through the EA ECG were used to synthesize a reference signal from an autoregressive model that considers these features and the heart rate to present a synthetic ECG signal that resembles its real counterpart [9].

### Redundant Denoising ICA Method

**Figure 2:**
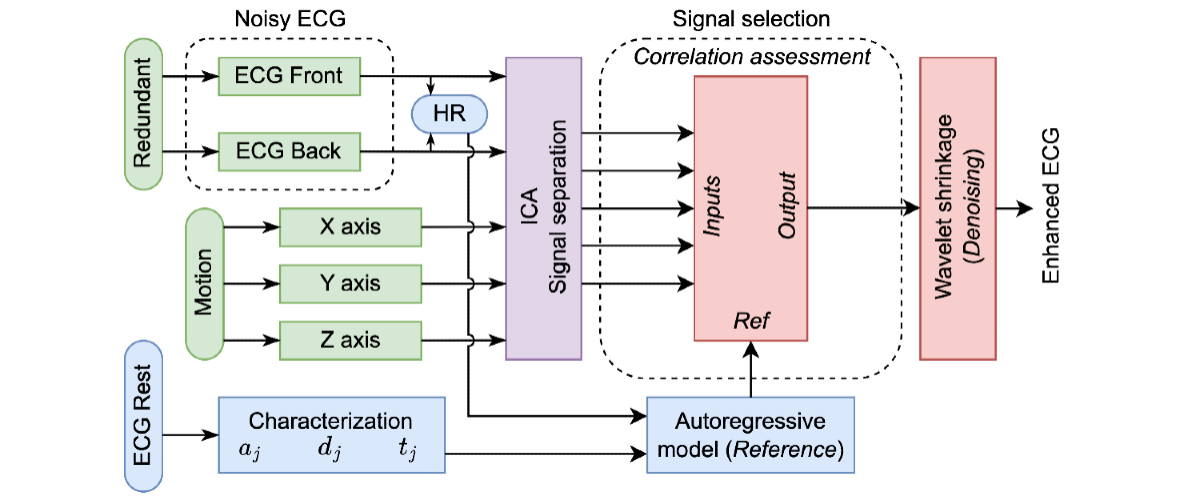
Block diagram representing the proposed Redundant denoising Independent Component Analysis method. The method considers the simultaneous and redundant acquisition of the electrocardiogram (ECG) signal contaminated with motion artifacts (green), the signal characteristics determination and the reconstruction of a reference ECG signal from a resting ECG signal of the same volunteer (blue), the components separation (purple), and the selection of the improved ECG signal (watermelon). HR: heart rate; ICA: independent component analysis.

The method proposed in this paper is based on the simultaneous and redundant acquisition of the ECG signal, the movement of the person, the separation of multichannel components, and the selection of the improved signal through the comparison with a modeled signal from previous information. The processing scheme of the Rd-ICA method is presented in Figure 2.

#### Acquisition of Redundant ECG and Motion Signals

The first step of the method consists in the acquisition of the redundant and simultaneous ECG signals and the movement signals of the person. The acquisition of the ECG signals in leads DI, DII, and DIII was performed both in the chest and back of the volunteers [24]. Redundancy of the signals is achieved by acquiring the leads of the ECG signal in the chest and back of the volunteer [53]. The acquisition in the chest and back is carried out simultaneously so the leads DI, DII, and DIII acquired in both areas are synchronized in time. The volunteers’ ECG segments acquired at rest were analyzed by a cardiologist to validate that the volunteers did not present evident cardiac pathology before analysis, confirming that the parameters of the signal segments are within the normal range in physiological terms.

**Figure 3:**
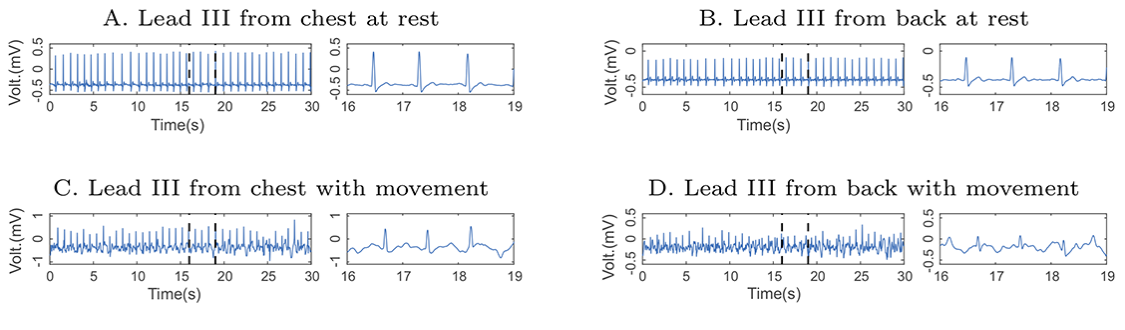
Epochs of 30-second lead III electrocardiogram (ECG) acquired in the chest and back of healthy volunteers at rest and with motion artifacts. The left panel in 4 axes shows 30-second epochs, while the right one shows a 3-second detail of the ECG signals. (A) Lead III from chest at rest, (B) lead III from back at rest, (C) lead III from chest with movement, and (D) lead III from back with movement.

Some of the ECG signals acquired from a healthy volunteer used in this work are presented (Figure 3). Figure 3A and Figure 3B show the ECG signal acquired at rest in the chest and back of the volunteer, respectively. Figure 3C and Figure 3D show the ECG signal with MA acquired in the chest and back of the volunteer, respectively, while the volunteer performed movement. Each figure has a sample of 30 seconds and a detail of 3 seconds to show the waveform. In addition, the synchronization between the signals acquired in the volunteer’s chest and back was presented, as there is no lag in the QRS complexes of each pair of signals.

The electrode movement pattern was acquired by adding an IMU on each electrode. That information was acquired simultaneously with the ECG signals, thus increasing the amount of information available for multichannel signal analysis. For the ECG signals contaminated with MA, the time that elapses between 2 consecutive R peaks was measured. This measurement represents the heart rate of the ECG signal during the movement. This feature of the signal was used to synthesize the reference ECG signal.

To calculate the heart rate, it is necessary to measure the time between 2 consecutive heartbeats. It is common to identify the QRS complex and measure the time elapsed between 2 of them consecutively. This method was used to calculate the heart rate, both in the signals acquired at rest and while moving (equation 3).



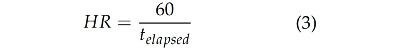



#### Modeling the Reference ECG Signal

To perform the comparison and selection of the resulting components after the multichannel analysis, a reference ECG signal was modeled from an autoregressive model that considers the features of the ECG signal segments such as amplitude, duration, and time of appearance of each segment [9,54]. It should be noted that the synthesized signal from an autoregressive model is only used as a comparison reference to determine which of the components obtained in the Rd-ICA method contains the highest percentage of information on the physiological signal of interest.

Synthesis of the reference ECG signal is based on the modeling of a group of functions *S*_0_(*t*), which represent each of the segments of the ECG signal as a modified waveform of the *S_j_*(*t*), which is obtained through Fourier models in the time interval (0≤t≤*T*) [9]. This time interval corresponds to the time between 2 consecutive R peaks that the modeled signal must have and is taken from the measured heart rate of the signal contaminated with MA. The mathematical equation describes the construction of each segment of the reference ECG signal (equation 4).


S_0_(t) = a_j_S_j_(d_j_t + t_j_) + c_j_; a_j_>0,d_j_>0 (4)

Where *a_j_*, *d_j_*, *t_j_*, and *c_j_* are the coefficients of amplitude, duration, time of appearance, and offset of the signal, respectively. If the variation of the baseline is subtracted in the processing step, *c_j_* can be omitted.

The autoregressive model requires the definition of the features of each segment of the signal (P, Q, R, S, T, and U) and the heart rate that the modeled signal will have. The features of amplitude, duration, and time of appearance of each segment were obtained from applying the EA ECG method to a signal previously obtained during the volunteer’s rest [16]. The EA ECG method allows to characterize the waveform of the ECG signal acquired at rest and to extract the coefficients for the synthesis of the reference ECG signal (equation 4). This later has the waveform of the ECG signal at rest, which is free of artifacts but includes the heart rate of the ECG contaminated with MA.

#### Separation and Selection of Multichannel Sources

With the premise that the interest information of the ECG signal comes from an independent source, which is the heart, and the MA come from sources other than this; the redundant measurement of the ECG signal was used to obtain information from a single source in 2 different sensors. Each of these sensors acquires information from artifacts from different sources. We assumed that redundant signals will have a common component that will be the ECG lead that is being measured in the chest and back of the volunteer and will have independent components from different sources of MA.

To identify the relative movement of the volume conductor, a set of signals formed by the redundant evaluations of an ECG derivation acquired in the volunteer’s chest and back is obtained as well as the movement signals in 3 orthogonal axes obtained with the IMU.

In this regard, the ICA method was used to perform an analysis of the signal data set to identify common information between the different sensed channels and to separate it from independent sources, using the ICA model.

**Figure 4:**
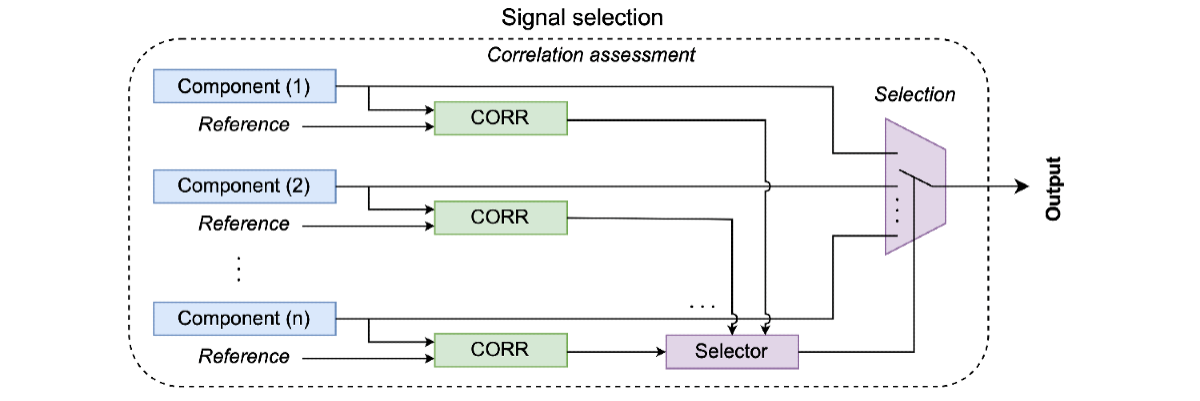
Selection method for the component that contains the greatest amount of information of the electrocardiogram signal source. CORR: correlation.

Once the independent components were obtained, the component with more information about the ECG signal was determined. For this, the reference ECG signal was used that was synthesized from the features of the signal acquired at rest from the same volunteer, the heart rate measurement from the ECG signal with MA and the autoregressive model that considers the characteristics and heart rate [9]. Figure 4 presents the assessment and selection method of different components with ECG information.

Each component resulting from the ICA method is compared with the reference signal through the correlation method (Figure 4), which provides a quantitative measure of the similarity between 2 signals. After that, the selection of the component with the highest correlation with the reference ECG signal is made. This component is processed in the final stage of filtering.

#### Final Stage of ECG Signal Filtering

Once the component with the most ECG information is obtained, it is filtered and improved with the WS method. With this method, noise filtering of components of the signal with high and low frequency is performed to obtain an improved ECG signal.

### Validation and Assessment of the Rd-ICA Method

#### Overview

To validate the Rd-ICA method, the performance presented by it is evaluated and compared with that of other state-of-the-art denoising methods such as the WS and WICA methods [16].

As presented earlier, the WS and WICA methods were applied only to the leads acquired on the chest of the volunteers, as each of these methods proposes, and the performance presented by these methods was measured. On the other hand, the Rd-ICA method was applied to the same set of signals but included the redundant signals and motion signals as proposed. The performance of this method was also measured.

The performance measurement presented by these methods was performed through the calculation of indexes traditionally used in signal processing. These indexes were the SNR, DTW, cross-correlation, and the measurement of the difference percentage of the signals’ features through the EA ECG method with respect to an ECG signal acquired at rest from the same volunteer. For this, an index that considers the distortion introduced by the denoising methods when performing the signal improvement was proposed. This index was called WDA.

#### Signal-to-Noise Ratio

The SNR was used to quantify the improvement of the enhanced signal after the denoising methods were applied. The SNR reflects the difference between input (reference signal) and output (enhanced signal) of the specific denoising methods (equations 5, 6, and 7) [55,56].



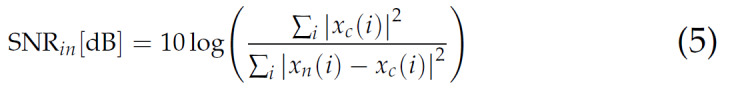





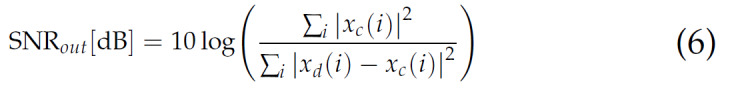





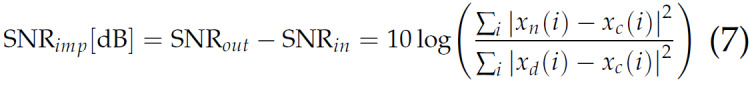



Where *x_c_* is the clean ECG signal, *x_n_* denotes the noisy ECG, and *x_d_* represents the denoised ECG.

#### Dynamic Time Warping

The DTW method allows to calculate the minimum Euclidean distance between each sample of the signal to be compared *S_j_*(*t*) and each point of the reference signal *S*_0_(*t*) [57,58]. The method uses 2 matrices of identical size to perform the calculation. Matrix *S*1_m×n_ contains *m* copies of the reference signal *S*_0_(*t*)_1×_*_n_* in the rows, and matrix *S*2_m×n_ contains *n* copies of the signal to compare *S_j_*(*t*)_m×1_ in the columns [17]. The distance matrix *D*_m×n_ is calculated using the single dimension Euclidean distance as shown in equation 8.



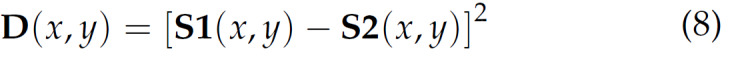



Where 1 ≤ *x* ≤ *m* and 1 ≤ *y* ≤ *n*. Starting in position (1,1) of *D*, a cost matrix *C* is created to store the accumulated distance of the previous column and row, which are calculated with equation 9.



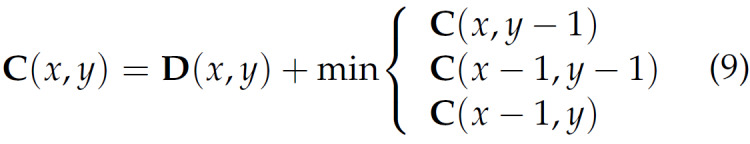



The path of minimum distance is found from cost matrix *C*, starting at the position (*m,n*) of the matrix and moving toward the adjacent position of lowest cost until reaching the beginning. These positions are saved and will then be identified in matrices *S*_1_ and *S*_2_ to create the minimum difference aligned signals *S*_1_*_w_* and *S*_2_*_w_*, respectively. In this process, it is possible that some samples of the matrix *S*_1_ or *S*_2_ are repeated to conform to the vectors *S*_1_*_w_* and *S*_2_*_w_*, which is an index of the difference between both signals and the distortion of the evaluated signal. Through this method, the distance between the standard waveform of the modeled reference ECG signal at rest and the enhanced ECG signal is determined.

#### Cross-Correlation

Cross-correlation is a measure of similarity between 2 signals. This is measured from the displacement and the superposition of one signal on the other to determine the level of similarity between both. This is known as sliding dot product and is defined in equation 10.



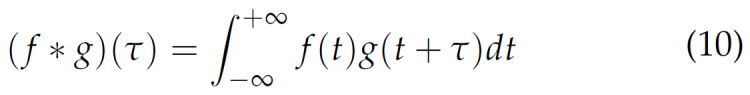



In this work, cross-correlation was used as an index to determine the performance of the denoising methods. Similarity between the reference ECG signal and the enhanced ECG signal was evaluated through the cross-correlation index.

#### Weighted Distortion Assessment

For the calculation of the WDA index, the percentage similarity vector of the features (△EA) and (△W) and the vector of weighted weights for the features are defined. These 2 vectors are defined by equations 11 and 12.


△EA = [a_P_, d_P_, t_P-R_, a_R_, d_R_, t_R-T_, a_T_, d_T_] (11)

△W = [w_P1_, w_P2_, w_P3_, w_R1_, w_R2_, w_R3_, w_T1_, w_T2_] (12)

*a*_P_, *d*_P_, *t*_P-R_, *a*_R_, *d*_R_, *t*_R-T_, *a*_T_, *d*_T_ corresponds to the similarity percentages of each feature obtained from the EA ECG. These are calculated using equation 13 [16]. *w*_P1_, *w*_P2_, *w*_P3_, *w*_R1_, *w*_R2_, *w*_R3_, *w*_T1_, *w*_T2_ correspond to the weights to define the relevance that each feature will have on the calculation of the WDA index. The WDA index is described in equation 14.

Similarity = (100% − %difference) / 100 (13)

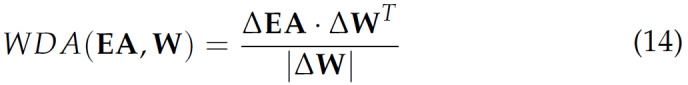



The coefficients’ values of the weight vector (△W) must be chosen between 0 and 1. These represent the percentage of relevance given to the preservation of a certain feature in the WDA performance index assessment. In this study, 4 different cases were evaluated: (1) all features have equal weight, so all the coefficients in the vector (△W) will be equal to 1; (2) the amplitude of the P wave will be more relevant in the analysis, thus *w*_P1_=0.9 and the other coefficients will have a weight of 0.5; (3) the amplitude of the QRS complex will be more relevant, *w*_R1_=0.9, and the other coefficients will have a weight of 0.5; and (4) the amplitude of the T wave will be more relevant, *w*_T1_=0.9, and the other coefficients will have a weight of 0.5. A high value of the WDA index indicates that the denoising was performed satisfactorily and the distortion introduced by the denoising methods was low, also indicating a better conservation of the features of the ECG signal.

### Ethics Approval

The study was conducted according to the guidelines of the Declaration of Helsinki and approved by the Human Studies Institutional Ethics Committee of Universidad de Antioquia (protocol code 16-59-711 of May 19, 2016). Informed consent was obtained from all participants involved in the study.

## Results

This section shows the results of the Rd-ICA method application for the reduction of MA in ECG signals and the assessment and comparison of the Rd-ICA method performance with the WS and WICA methods applied on ECG signals acquired from healthy volunteers in rest and movement. The performance evaluation of the Rd-ICA method was conducted through the measurement of indexes such as SNR, DTW, and the WDA index proposed in this paper based on the measurement of features from the EA ECG.

### EA ECG of the ECG Signal at Rest and Movement

The EA ECG method was applied to ECG signals of volunteers acquired in resting conditions (Figures 3A and 3B) and to ECG signals acquired with MA (Figures 3C and 3D).

**Figure 5:**
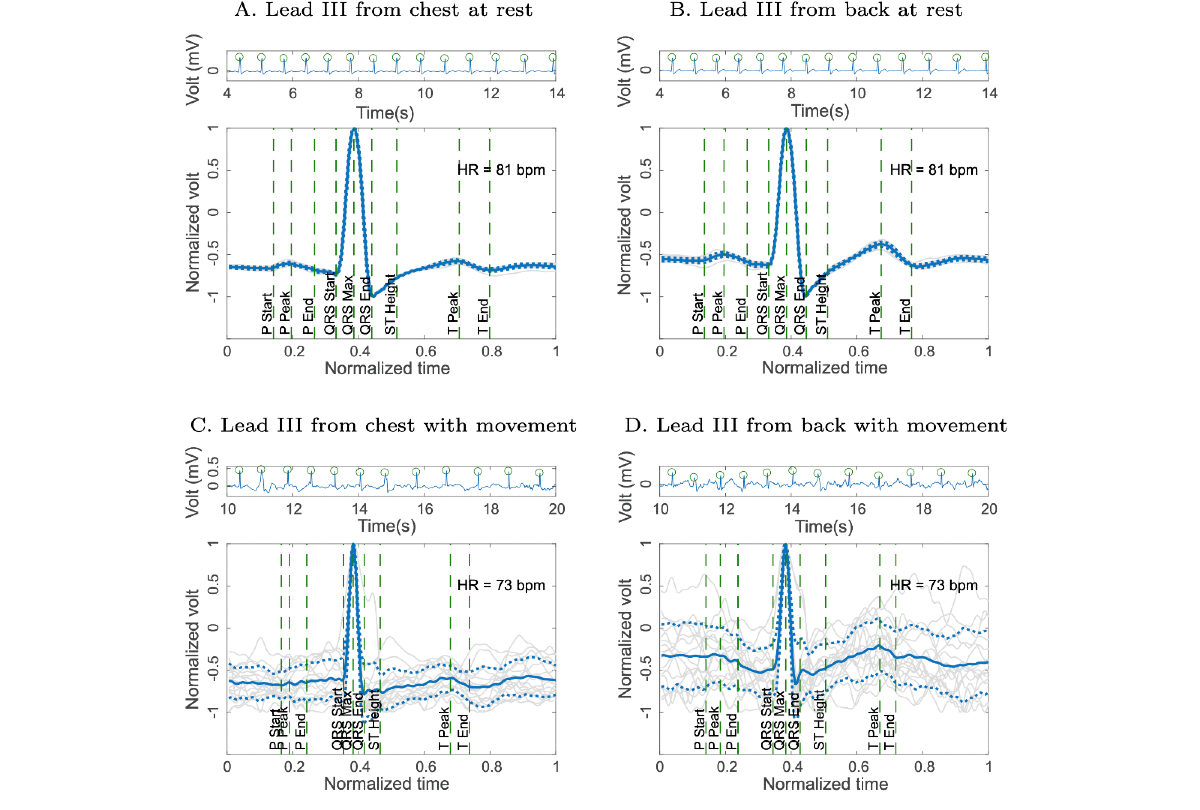
Ensemble average (EA) electrocardiogram (ECG) for ECG signals acquired at rest and movement, on the chest and back of the volunteer. The continuous centerline represents the EA ECG of the signal while the dashed lines represent the SD of the EA ECG. (A) Lead III from chest at rest, (B) lead III from back at rest, (C) lead III from chest with movement, and (D) lead III from back with movement. HR: heart rate; P: P segment; Q: Q segment; QRS: QRS complex; ST: ST interval; T: T segment.

Figure 5A and Figure 5B show the EA ECG of the signals acquired in resting conditions in the chest and back of a volunteer, respectively. The solid line represents the average signal and the dashed lines represent the SD signals. Figures 5C and 5D present the acquired signals in movement conditions; in the same way it presents the average signal by means of the continuous line and the SDs by means of the dashed lines. The dispersion of the different waves that compose the EA is observed during the movement.

### Application of Denoising Methods

Some state-of-the-art denoising methods were applied to ECG signals contaminated with MA. These signals were selected only from the chest of the volunteers while they performed movement. The methods applied were the WS and WICA method.

**Figure 6:**
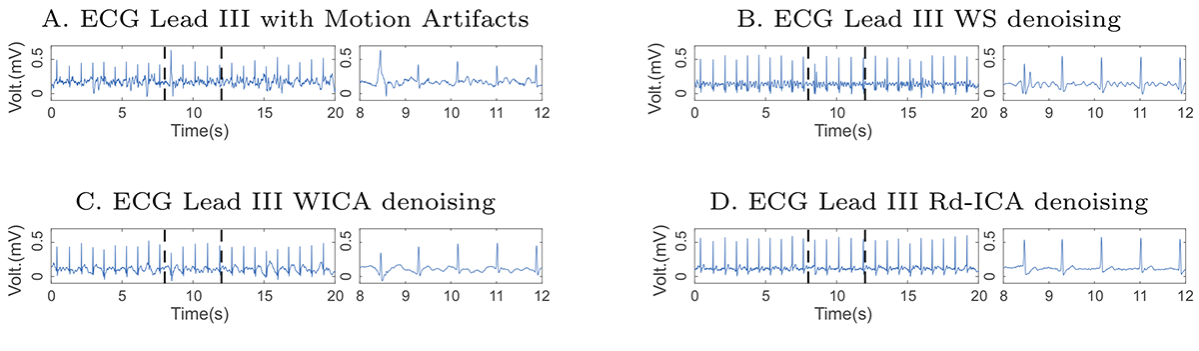
Epochs of 30-second lead III electrocardiogram (ECG) signal contaminated with motion artifacts (MA) after denoising and enhancement. The left panel in 4 axes shows 30-second epochs, while the right one shows a 4-second detail of the ECG signals. (A) ECG lead III with MA, (B) ECG lead III with WS denoising, (C) ECG lead III with WICA denoising, and (D) ECG lead III with Rd-ICA denoising. Rd-ICA: Redundant denoising Independent Component Analysis; WICA: wavelet independent component analysis; WS: wavelet shrinkage.

**Figure 7:**
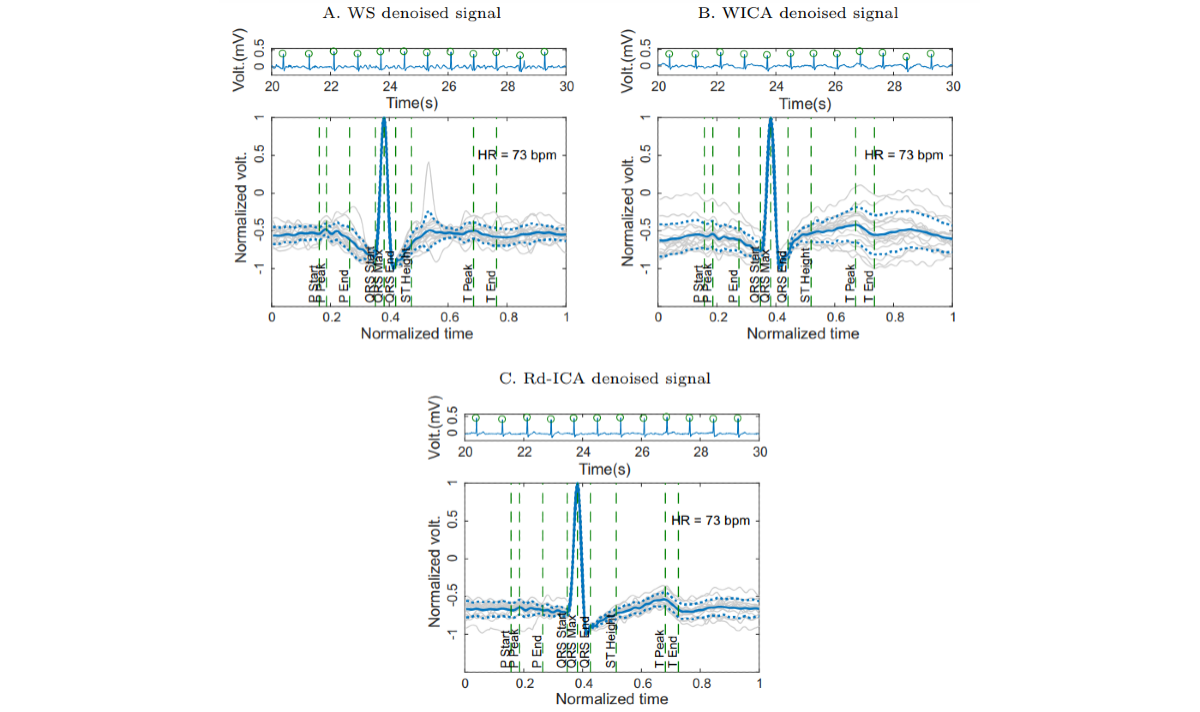
Ensemble average (EA) electrocardiogram (ECG) for enhanced ECG signals through the wavelet shrinkage (WS), wavelet independent component analysis (WICA) and Redundant denoising Independent Component Analysis (Rd-ICA) methods. The continuous centerline represents the EA ECG of the signal while the dashed lines represent the SD. (A) ECG lead III with WS denoising, (B) ECG lead III with WICA denoising, and (C) ECG lead III with Rd-ICA denoising. HR: heart rate; P: P segment; Q: Q segment; QRS: QRS complex; ST: ST interval; T: T segment.

The Rd-ICA method was applied to the redundant and simultaneous ECG signals contaminated with MA and acquired in the volunteer’s chest and back. The comparison was made with a reference ECG signal synthesized from an autoregressive model. The result of applying denoising methods on the ECG signal of a volunteer is presented (Figure 6). In addition, the EA ECG of the denoising result of the ECG signal through the WS, WICA, and Rd-ICA methods is shown (Figure 7).

A graphic comparison between the results of the denoising presented by each of the evaluated methods is presented (Figure 6). In addition, the result of applying the EA ECG over the improved signals with each of the methods, including average and SD signals is presented (Figure 7).

### Validation and Assessment of the Rd-ICA Method

#### SNR Results

To determine the performance of the denoising methods, the improvement SNR (SNR_imp_) was evaluated on the enhanced ECG signals (Figure 6). The SNR_imp_ was calculated using equation 7 as the difference between the SNR_in_ measured on the ECG signal contaminated with MA before enhancement and the SNR_out_ measured on the enhanced ECG signal through each method. The SNR_in_ obtained for the signal contaminated with MA was −4.64 (SD 0.43). A SNR_out_ of −3.94 (SD 0.35) for WS, −3.22 (SD 0.34) for WICA, and −1.07 (SD 0.15) for Rd-ICA was obtained. The above represents a SNR_imp_ of 0.70 (SD 0.37) for WS, 1.42 (SD 0.31) for WICA, and 3.58 (SD 0.36) for the Rd-ICA method. A larger SNR_imp_ was observed in the Rd-ICA method, followed by the WICA method and finally by the WS method.

#### DTW Results

Through the DTW method, it was possible to identify the distance percentage between a waveform of a reference signal with the waveform of a signal under evaluation. This percentage was calculated from the DTW method [57]. The lower the percentage, the greater the similarity between the evaluated signal and the reference signal.

The DTW showed 42.62% (SD 8.64%) of difference between MA contaminated signal and the reference signal. Distance percentages of 41.32% (SD 8.01%) for WS, 44.23% (SD 16.31%) for WICA, and 19.72% (SD 6.25%) for Rd-ICA were obtained. A smaller percentage of distance was obtained in the result of the Rd-ICA method. For the WICA and WS methods, an increase in the distance percentage was observed with respect to the signal contaminated with MA, which suggests a distortion increase.

#### Cross-Correlation

Cross-correlation provides information about the similarity between 2 signals. In this case, the similarity between the reference ECG signals obtained from the model and the enhanced ECG signals was evaluated. For ECG signal contaminated with MA it was obtained a cross-correlation of 6.08 (SD 1.48). Cross-correlation of 5.74 (SD 0.67) for WS, 5.84 (SD 1.31) for WICA, and 8.66 (SD 0.39) for Rd-ICA were obtained.

#### WDA and Difference Percentage

From the EA ECG, the features of the resting ECG signals, ECG signals contaminated with MA, and the enhanced ECG signals were measured. The difference percentage of the features between the contaminated and enhanced ECG signals relative to the signal acquired at rest was measured [16]. Table 1 shows the difference percentage for each ECG signal feature through the EA ECG method. The average and SD values are presented.

The WDA index assessment was performed for 4 different cases:

1. All features with equal relevance level 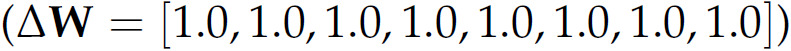.
2. Greater relevance of the P wave 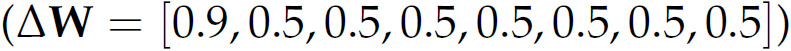.
3. Greater relevance of the QRS complex 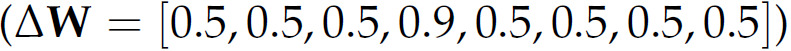.
4. Greater relevance of the T wave 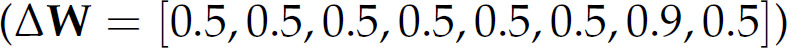.

Table 2 shows the result of the distortion analysis through the WDA index for signals contaminated with MA and enhanced through denoising methods.

Previous results show the method that presents the best performance in denoising and that preserves the signal waveform features with less distortion is the Rd-ICA method. This supports the results obtained from the other indexes evaluated.

**Table 1:** Difference percentage between the features of enhanced electrocardiogram signals and electrocardiogram signals acquired at rest.

Difference	Movement, mean (SD)	Wavelet, mean (SD)	WICA^a^, mean (SD)	Rd-ICA^b^, mean (SD)
Amplitude P	52.56 (23.20)	59.91 (25.06)	42.03 (36.77)	*27.46 (7.37)* ^c^
Duration P	25.86 (19.79)	14.83 (6.78)	30.19 (9.49)	*14.59 (7.58)*
Time P-QRS	30.63 (10.62)	26.99 (16.26)	20.56 (11.50)	*13.87 (8.37)*
Amplitude QRS	18.62 (4.87)	4.38 (1.85)	9.76 (8.61)	*3.24 (2.00)*
Duration QRS	11.29 (7.88)	6.37 (5.04)	9.62 (9.33)	*4.89 (2.58)*
Time QRS-T	40.58 (20.12)	31.05 (14.68)	33.70 (15.90)	*20.10 (7.91)*
Amplitude T	47.01 (19.61)	52.24 (43.02)	53.36 (35.22)	*18.63 (5.53)*
Duration T	27.03 (9.86)	12.06 (8.07)	10.73 (7.80)	*11.54 (5.32)*
Average	31.70 (14.49)	25.88 (15.09)	26.24 (16.83)	*13.04 (5.83)*

^a^WICA: wavelet independent component analysis.

^b^Rd-ICA: Redundant denoising Independent Component Analysis.

^c^Italicized values indicate the best performance in the dynamic time warping assessment.

**Table 2:** Evaluation of the weighted distortion assessment (WDA) index from the enhanced electrocardiogram signals for 4 different cases of specific feature relevance.

WDA	Movement	Wavelet	WICA^a^	Rd-ICA^b^
Case 1	1.93	2.10	2.09	*2.35* ^c^
Case 2	1.74	1.85	1.91	*2.18*
Case 3	1.87	2.05	2.02	*2.26*
Case 4	1.76	1.88	1.87	*2.16*

^a^WICA: wavelet independent component analysis.

^b^Rd-ICA: Redundant denoising Independent Component Analysis.

^c^Italicized values indicate the best performance in the dynamic time warping assessment.

## Discussion

### Principal Findings

This paper presents a novel method for MA reduction in ECG signals through the acquisition of redundant and simultaneous signals, acquisition of the person’s movement, modeling of the reference signal from prior knowledge of the ECG signals at rest, and processing of this set of signals through multichannel processing techniques. This method was called Rd-ICA. This was applied to a data set composed of ECG signals acquired from healthy volunteers at rest and in movement conditions.

The performance of the proposed Rd-ICA method was compared with state-of-the-art methods for MA reduction such as WS and WICA. To compare the performance of the different methods, each was applied to the data set, and the performance was evaluated from the measurement of indexes such as SNR, DTW, cross-correlation, and the proposed WDA that consider the morphological features of the signal [16].

The use of a single denoising method does not guarantee the reduction of noise in all features. Sometimes it is necessary to use specific denoising methods to improve the signal and maintain some feature with fidelity. Despite this, the Rd-ICA method presents a good alternative for the reduction of MA in contaminated ECG signals, as shown by the results obtained in this paper.

### Comparison With Prior Works

The ECG signals acquired redundantly in the chest and back of the volunteers showed similar information from the same source; this information belongs to the ECG signal that is of interest in this study [59]. In addition, it contains information from other sources among which are MA, which are measured as information from independent sources. This allowed the separation of the signal of interest and the signal of artifacts through the proposed multichannel signal processing technique.

Most of the methods reported are based on the separation by components, the extraction of the noise components and their elimination, then the reconstruction of the signal of interest. The works at the frontier of knowledge make use of methods such as EMD, wavelet, and adaptive filters with some modifications and improvements, and these are the ones that have presented the best performance, but according to those reports, they also introduce a large amount of distortion in the signal of interest.

The method proposed in this paper is advantageous and novel from the point of view that it acquires the signal redundantly, thus providing a way to validate the processing applied to the signal of interest. Other finding was that both components of the ECG signal acquired on chest and back were significantly similar, while the artifacts contamination showed differences between the 2 signals acquired on the chest and back of volunteers [24]. In addition, the motion component helps to determine the dynamics of the signal, evaluate the nonlinearity of contamination by artifacts and perform a better estimation of the components of interest through the proposed Rd-ICA method.

### Application Spectrum

This technique presents its application with wearable vital signs monitoring devices, which have their main field of application in outpatient vital signs monitoring. This technique requires a modification in the signal acquisition hardware as it requires redundant and simultaneous ECG signal acquisition from the chest and back of patients. In addition, it requires the measurement of movement through IMUs. These modifications are possible to implement in wearable devices [31]; therefore, the technical feasibility can be affirmed for implementation in outpatient monitoring.

The proposed technique has important potential in the processing of physiological signals from different sources with the use of redundant acquisition of the interest signals. It shows its application in the identification of signals from cardiac arrhythmias in conjunction with the EA ECG method and the proposed WDA index. Some of the ECG monitoring devices that potentially may include this technique to reduce artifacts and to improve their diagnostic potential are external loop recorders, implantable loop recorders, traditional Holter, and wearable ECGs. This kind of improvement will reduce the associated costs with repeated tests.

The method proposed in this paper presents a considerable advance in the reduction of MA in ECG signals as the results showed an improvement in the denoised signal. Its advantage is not only in the evaluation of signal indexes such as SNR but also in the preservation of the signal waveform, low distortion introduction, and the potential use in medical diagnosis. It was observed that the Rd-ICA method presents a significant improvement in the conservation of the characteristics of the signal compared with the WS and WICA when it was evaluated using the EA ECG method.

In the same way, the possibility to have the redundant ECG signal and the motion signals from the inertial sensors provides the proposed method with the capability to separate the component of interest from the components of artifacts. At the same time, it allows the method to determine these components autonomously by comparing this signal with a reference signal built from an autoregressive model that considers the cardiovascular characteristics of the volunteer. This allows the proposed method to be implemented in wearable monitoring systems and in autonomous monitoring systems.

### Future Work

On the other hand, there is evidence of the need to carry out more extensive research to evaluate the reliability and clinical validity of this method in patients with relevant diseases. Although this method presents the possibility to differentiate events coming only from the cardiovascular system and separate them from external events such as the volunteer’s movement. This is due to the possibility of obtaining the ECG signals redundantly and simultaneously. In addition, the need to add new sensors for the redundant measurement of the signals and the obtaining of the movement signal through IMUs implies significant modifications in the hardware of the existing monitoring systems. Despite this, the possibility of making these modifications is evident and opens the possibility to generate new designs, enabling the growth of the market for wearable medical devices.

Currently, the research and development of biosensors have a great boom thanks to the advantages they show, such as easy application and portability. Their easy implementation in wearable devices for the continuous measurement of different vital sign signals or physiological variables in everyday environments makes these devices a great option in vital signs monitoring [60]. Despite its great applicability, the information acquired by these biosensors is distorted by different sources of noise and artifacts, such as MA. The method proposed in this work is not limited to the denoising of the ECG signal, but it can be used for other physiological signals that can be redundantly acquired and are susceptible to be affected by MA. Some of these sensors are PPG biosensors, enzymatic biosensors for glucose measurement, intraocular pressure, and hydration percentage [60,61].

### Conclusions

The technique’s ability to improve the quality of the signal is critical for diagnosing specific cardiac arrhythmias in real-world use. The diagnostic yield has been shown to be a major determinant in a technique’s economic assessment; for example, in diagnosis after palpitations [62] or syncopes [63,64], in screening of athletes [65,66], or in identifying asymptomatic atrial fibrillation [33,67]. To explore these applications, the acquisition of a database that considers more extreme movements and patients with common cardiac pathologies is required, which will provide information about the effect of artifact promotion techniques in the correct identification of arrhythmias or the malfunction of heart. Such a database would allow future work on the proposed method and a benchmark with existing methods to evaluate their performance in MA reduction as well as its benefits in the identification of waveforms modified by specific cardiac arrhythmias.

## Abbreviations

DTW: dynamic time warping
DWT: discrete wavelet transform
EA: ensemble average
ECG: electrocardiogram
EMD: empirical mode decomposition
ICA: independent component analysis
IMU: inertial measurement unit
MA: motion artifacts
PPG: photoplethysmography
Rd-ICA: Redundant denoising Independent Component Analysis
SNR: signal-to-noise ratio
WDA: weighted distortion assessment
WICA: wavelet independent component analysis
WS: wavelet shrinkage
